# Adaptive soft sensor based on transfer learning and ensemble learning for multiple process states

**DOI:** 10.1002/ansa.202200013

**Published:** 2022-06-10

**Authors:** Nobuhito Yamada, Hiromasa Kaneko

**Affiliations:** ^1^ Department of Applied Chemistry School of Science and Technology Meiji University Kawasaki Japan

**Keywords:** adaptive soft sensor, ensemble learning, locally weighted partial least squares, multiple grades, negative transfer, transfer learning

## Abstract

The objective of this study is to develop an adaptive software sensor technique that can predict objective process variables for a target grade in a plant while also considering information related to various other grades. We use a dataset of the target grade as the target domain and those of the other grades as source domains to perform transfer learning. Multiple models or sub‐models are constructed by setting a source domain for each grade and changing the number of samples used as the source domain. Furthermore, to prevent the negative transfer, the use of a source domain is automatically judged. In this study, we constructed sub‐models using the locally weighted partial least squares approach as an adaptive soft sensor technique. The values of an objective variable were predicted with ensemble learning using sub‐models. The effectiveness of the proposed method was verified using a dataset measured in an actual incineration plant, and the proposed method was able to accurately predict the product quality even when the plant was operated in five grades and when a new grade was produced.

**Abbreviations**
EL: ensemble learning
FEDA: frustratingly easy domain adaptation
JIT: just‐in‐time learning
LWPLS: locally weighted partial least squares
MW: moving window
RMSE: root‐mean‐squared error
SD: source domain
TD: target domain
TL: transfer learning

## 1 INTRODUCTION

Automatic process control systems have been introduced in various types of industrial plants to ensure their safe and stable operation. To rapidly control a process variable, it is necessary to continuously measure the value of the corresponding variable. Although process variables, such as temperature and pressure, can be measured in real‐time using hard sensors, the resulting product qualities (e.g. concentration and density) must be analysed in a laboratory. For this purpose, time‐consuming measurement protocols are required, which causes delays in the process control.

Thus, to estimate the values of process variables that are difficult to measure in real‐time, soft sensors are commonly employed.^1^ A soft sensor is a mathematical model (i.e. y = f(x)) that is constructed between process variables that are easy to measure (x) and process variables that are difficult to measure (y). By inputting the newly measured x values into the constructed model, the y values can be estimated in real‐time, thereby allowing rapid process control to be achieved.

In addition, some plants do not always produce a single product grade. Instead, they switch between multiple product grades or produce multiple grades at the same time; hence, soft sensors can be constructed using a dataset for each product grade of interest. However, the construction of a soft sensor for a new product grade, or for a grade that is not commonly produced, is a significant challenge as the number of available samples is small, or zero. To address these issues, the prediction accuracy can be improved by constructing a soft sensor using not only the corresponding datasets of the various known grades but also the datasets of other similar grades. Therefore, the analysis of time‐series data using transfer learning (TL)^2^ has attracted growing attention in recent years. In TL, when constructing a soft sensor for a target grade, the samples of the target grade are employed along with information obtained from samples of other grades, such as constructed models and optimized hyperparameters. During TL, the source samples are referred to as the source domain (SD) and the target samples are known as the target domain (TD).

Various time series data analyses using TL have already been conducted, including those based on extreme learning machine,^3^ long short‐term memory networks^4^ and TL with incremental learning.^5^ During these analyses, an enhanced prediction accuracy was achieved by transferring the information of other time‐series data to the target time series data. For example, Liu et al. developed a domain‐adaptation extreme learning machine to construct soft sensors for multiple processes, wherein only small samples are produced or available.^6^ In addition, Alakent proposed the combination of a moving window (MW)^6^ and just‐in‐time learning (JIT)^7^ using transductive inference,^8^ while they later proposed a combination of a task‐transferred JIT model with an MW learner in a transductive learning setting.^9^

Negative transfer,^10^ which is a phenomenon in which the prediction accuracy is reduced by transferring information, should be considered in TL. More specifically, the negative transfer is an important issue in TL and is considered to occur when the SD is completely unrelated to the TD, and when training in SD interferes with training in TD. In addition, when transferring information from the SD to the TD, only the information that is considered relevant to the TD should be transferred, rather than all the information collected for SD.^11^ In this context, Liu et al. proposed a method to calculate the distances between samples in the TD and samples in the SD. Subsequently, they selected samples in the SD and calculated the weight for each sample.^12^

Furthermore, it should be noted that more than one SD can exist in TL; for example, products of multiple grades are produced in a plant. In such cases, the use of multiple SDs is superior to that of a single SD in terms of prediction accuracy and model operation.^13^ In addition, Ye et al. constructed extreme learning machines using multiple time‐series datasets and integrated the predicted y values,^14^ while Xiao et al. used an analogue complexing method to calculate the similarity between the most recent sample group of a query sample and a past sample group, and then calculated the weights to integrate the predicted y values.^15^

The prediction accuracy of soft sensors decreases owing to changes in the process conditions, such as changes in the feedstock, catalyst degradation and fouling of the pipes. Therefore, adaptive soft sensors^16^ that utilize newly measured samples generated in a plant are used. More specifically, the newly measured sample is added to the training data and the soft sensor model is reconstructed. In this study, we focus on JIT‐type adaptive soft sensors, in which the model is constructed and a y value is predicted by weighting training samples based on the similarity between a query sample and the training data.

In the context of TL for adaptive soft sensors, the transfer of SD should be stopped from the viewpoint of negative transfer after the TD samples are accumulated to a certain extent; however, there are no criteria for determining when to stop TL. In addition, there have been no discussions related to the use of TL in adaptive soft sensors when there are multiple SDs.

Thus, we herein report the development of an adaptive soft sensor that utilizes the TL in the presence of multiple process states. We use multiple SDs according to the process states and set a threshold for the number of samples in the TD to stop TL for each SD. For each SD, when the number of samples in the TD is less than the threshold, an adaptive soft sensor is constructed using the TL. On the other hand, the number of samples in the TD exceeds the threshold, and an adaptive soft sensor is constructed with only the TD and without TL. Furthermore, to handle adaptive soft sensors according to multiple SDs, ensemble learning (EL) is introduced, and the y values are predicted by integrating adaptive soft sensors. The proposed method is expected to improve the prediction accuracy of soft sensors by using SDs and by combining TL and EL. For the purpose of this study, we use frustratingly easy domain adaptation (FEDA)^17^ as a TL technique and locally weighted partial least squares (LWPLS) as a JIT‐type adaptive soft sensor. The effectiveness of the proposed method over the traditional methods is verified using data measured in an actual plant. Finally, three case studies are conducted: (1) Under the assumption that products are produced while switching grades, (2) a grade that has been produced in small quantities in the past is produced again and (3) a new grade that has never been produced in the past is produced.

## 2 METHODS

### 2.1 Frustratingly easy domain adaptation

**FIGURE 1:**
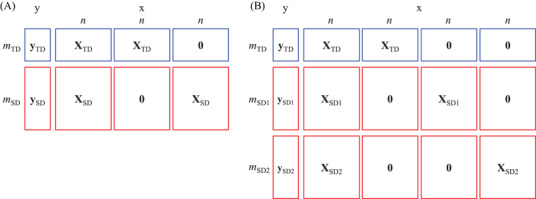
Basic concept of frustratingly easy domain adaptation (FEDA) used in this study. (A) One source domain (SD) and (B) two SDs

FEDA is a type of domain adaptation in TL that is easier to implement in any regression method, including adaptive soft sensor techniques, and it is also able to handle multiple SDs. Figure 1 shows the basic concept of the FEDA. Thus, when one SD exists, such as in the case shown in Figure 1A, the new datasets **X** ∈ R^(^ *^m^* ^TD+^ *^m^* ^SD)×(3^ *^n^* ^)^ and **y** ∈ R^(^ *^m^* ^TD+^ *^m^* ^SD)×1^ can be expressed as follows:

(1) $$X=\left(\begin{array}{ccc} X_{\mathrm{TD}} & X_{\mathrm{TD}} & 0\\ X_{\mathrm{SD}} & 0 & X_{\mathrm{SD}} \end{array}\right).$$

(2) $$y=\left(\begin{array}{c} y_{\mathrm{TD}}\\ y_{\mathrm{SD}} \end{array}\right),$$

where **X**_T_ ∈ R*^m^* ^TD×^ *^n^* and **y**_T_ ∈ R*^m^* ^TD^ are the TDs of x and y, respectively; **X**_SD_ ∈ R*^m^* ^SD×^ *^n^* and **y**_SD_ ∈ R*^m^* ^SD^ are the SDs of x and y, respectively; *m*_TD_ and *m*_SD_ are the number of samples in a TD and in an SD, respectively; and *n* is the number of variables in x. A regression model was constructed between the new datasets, **X** and **y**. The relationship between x and y that is common to both the TD and the SD is trained in the x‐variables of **X**_TD_ and **X**_SD_, while the relationship that is specific to the TD is trained in the x‐variables of **X**_TD_ and **0**, and the relationship specific to the SD is trained in the x‐variables of **0** and **X**_SD_.

When two SDs exist, such as in the case presented in Figure 1B, the new datasets **X** ∈ R^(^ *^m^* ^TD+^ *^m^* ^SD1+^ *^m^* ^SD2)×(4^ *^n^* ^)^ and **y** ∈ R^(^ *^m^* ^TD+^ *^m^* ^SD1+^ *^m^* ^SD2)×1^ are given as follows:

(3) $$X=\left(\begin{array}{cccc} X_{\mathrm{TD}} & X_{\mathrm{TD}} & 0 & 0\\ X_{\mathrm{SD}1} & 0 & X_{\mathrm{SD}1} & 0\\ X_{\mathrm{SD}2} & 0 & 0 & X_{\mathrm{SD}2} \end{array}\right),$$

(4) $$\left(\begin{array}{c} y_{\mathrm{TD}}\\ y_{\mathrm{SD}1}\\ y_{\mathrm{SD}2} \end{array}\right),$$

where **X**_SD1_ ∈ R*^m^* ^SD1×^ *^n^* and **y**_SD1_ ∈ R*^m^* ^SD1^ are the first SDs of x and y, respectively; **X**_SD2_ ∈ R*^m^* ^SD2×^ *^n^* and **y**_SD2_ ∈ R*^m^* ^SD2^ are the second SDs of x and y, respectively; and *m*_S1_ and *m*_S2_ are the number of samples in the first and second SDs, respectively. A regression model was constructed between the new datasets, **X** and **y**. The relationship between x and y that is common to the TD, the first SD and the second SD is trained in the x‐variables of **X**_TD_, **X**_SD1_ and **X**_SD2_, respectively. In addition, the relationship that is specific to the TD is trained in the x‐variables of **X**_TD_, **0** and **0**, and the relationship that is specific to the first SD is trained in the x‐variables of **0**, **X**_SD1_ and **0**. Furthermore, the relationship specific to the second SD is trained in the x‐variables of **0**, **0** and **X**_SD2_.

Notably, this method can be extended in the same manner when there are more than three SDs.

### 2.2 Proposed method

**FIGURE 2:**
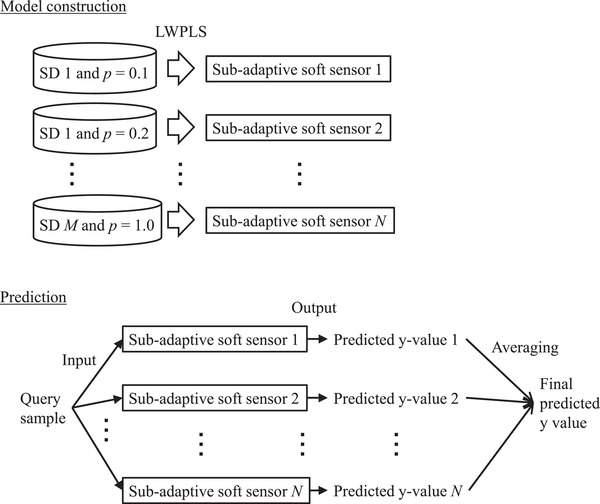
Basic concept of the proposed method

Figure 2 illustrates the basic concept of the proposed method. More specifically, in the proposed method, when using FEDA to construct a model with training data, a number of samples were selected for each SD and then combined with the TD to construct an LWPLS model. This model is called a sub‐adaptive soft sensor. Each time a query sample is obtained, each sub‐adaptive soft sensor is reconstructed and a y value is predicted. In addition, for each sub‐adaptive soft sensor, the decision to use or not to use FEDA is made, and model reconstruction and prediction are performed. The specific flow of the construction and prediction of the sub‐adaptive soft sensor can be summarized as follows. The ratio of *m_i_*, which is the number of samples in the *i*th SD to the number of samples in the SD, is *p* for each SD, and *p* is set to 0.1, 0.2, …, 0.9 and 1.0. When there are more samples in the TD than *m_i_* ×*p*, an LWPLS model is constructed with only the samples of the TD to predict the y value for a query sample. When there are fewer samples in the TD than *m_i_* ×*p*, the Euclidean distance (x) between each sample in the *i*th SD and all samples in the TD is calculated in each case. The *m_i_* ×*p* samples are selected in the order of decreasing average distance, and an LWPLS model is constructed using FEDA to predict the y value for a query sample. Accordingly, when the number of SDs is *M* and the number of *p* candidates (0.1, 0.2, …, 0.9 and 1.0) is 10, 10*M* sub‐adaptive soft sensors are constructed. Because various models are constructed with different SDs and different numbers of samples in the corresponding SDs, the proposed soft sensor can adapt to a variety of process states. The final predicted y value is the average of the 10*M* predicted y values in EL.

## 3 RESULTS AND DISCUSSION

To verify the effectiveness of the proposed method, we used a dataset from an incinerator plant at Sumitomo Chemical Co., Ltd. In an incinerator, products are produced by switching the grades, where y is the product quality and indicates the degree of firing, and x represents 51 process variables, such as the temperature and pressure. For the purpose of this study, the following three case studies were conducted:

1. Case study 1: Production of products while switching their grades;
2. Case study 2: Production of products of a grade that has been produced in small quantities in the past; and
3. Case study 3: Production of new‐grade products that have never been produced in the past.

To compare the proposed methods, LWPLS using all SDs (SD‐LWPLS), LWPLS using only the TD (TD‐LWPLS) and LWPLS with FEDA for all SDs (FEDA‐LWPLS) were adopted. The number of components and the similarity coefficient in LWPLS were determined to maximize the determinant coefficient *r* ^2^ after five‐fold cross‐validation. The LWPLS program was written in Python.^18^

### 3.1 Case study 1: production of products while switching their grades

In this case study, the y values were predicted using adaptive soft sensors in a situation in which products were produced while switching their grades. A total of 138 groups of five grades were selected, with one group representing each period of continuous production (from the time of grade switching to the time of the subsequent grade switching). The samples from the first group for each grade were used as training data, while the samples from the other groups were used as test data.

Tables 1 and 2 show the *r* ^2^ and root‐mean‐squared error values (RMSE, which is the average of y errors) of the test data for each grade. More specifically, the calculated values for the SD‐LWPLS, TD‐LWPLS and FEDA‐LWPLS methods, in addition to the proposed method, are shown. Although *r* ^2^ was negative in grades 1, 2 and 3 for the SD‐LWPLS, TD‐LWPLS and FEDA‐LWPLS methods, the proposed method was able to accurately predict the y values for all grades. In the case of the TD‐LWPLS method, *r* ^2^ was slightly higher than that of the proposed method for grade 4, while the *r* ^2^ value of the proposed method was significantly higher for the other grades, and *r* ^2^ became negative for grade 2 in the TD‐LWPLS method. In addition, the RMSE values shown in Table 2 indicate that the prediction errors of the proposed method are lower than those of the traditional methods. Compared with the TD‐LWPLS method, the proposed method can reduce the average y error by up to 31%, and so it was confirmed that the proposed method exhibited a superior prediction accuracy compared to the traditional methods.

**TABLE 1:** Obtained *r* ^2^ values for the test data of the various grades used for product generation (case study 1)

	**SD‐LWPLS**	**TD‐LWPLS**	**FEDA‐LWPLS**	**Proposed method**
Grade 1	−0.6	0.261	−0.05	0.344
Grade 2	0.182	−0.02	0.114	0.518
Grade 3	−0.05	0.231	−0.2	0.356
Grade 4	0.196	0.410	0.318	0.407
Grade 5	0.003	0.385	0.152	0.416

Abbreviations: FEDA, frustratingly easy domain adaptation; LWPLS, locally weighted partial least squares; SD, source domain; TD, target domain.

**TABLE 2:** Root‐mean‐squared error values (RMSE) for the test data of the various grades used for product generation (case study 1)

	**SD‐LWPLS**	**TD‐LWPLS**	**FEDA‐LWPLS**	**Proposed method**
Grade 1	1.469	1.011	1.204	0.952
Grade 2	2.545	2.848	2.648	1.953
Grade 3	2.510	2.147	2.691	1.965
Grade 4	3.614	3.096	3.329	3.106
Grade 5	2.983	2.342	2.750	2.282

Abbreviations: FEDA, frustratingly easy domain adaptation; LWPLS, locally weighted partial least squares; SD, source domain; TD, target domain.

In the traditional methods, it was not possible to determine in advance whether the prediction accuracy of the TD‐only model or that of the model using TL was superior for the test data, which could account for the large y errors. However, the proposed method was able to handle both situations through the use of the models with only the dataset of a target grade and the models using TL. As a result, the y errors were reduced using the EL approach.

### 3.2 Case study 2: production of products of a grade that has been produced in small quantities in the past

In this case study, the y values were predicted using adaptive soft sensors. Although the number of training data for the target grade was ≤22, the datasets of the other five grades in case study 1 were used as SDs. The number of test data points for the target grade was 94.

**FIGURE 3:**
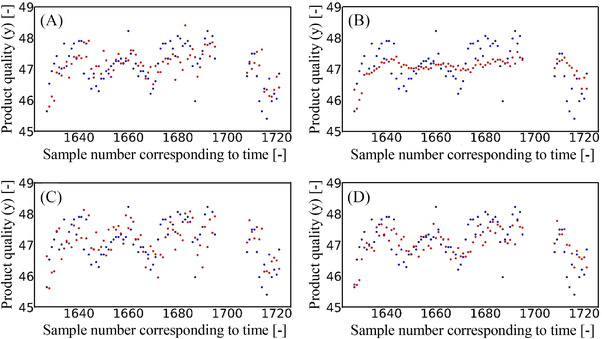
Variation in *y* overtime during the generation of products of a grade that has been produced in small quantities in the past (case study 2). The blue and red points represent the actual and predicted *y* values, respectively. (A) source domain–locally weighted partial least squares (SD‐LWPLS), (B) target domain (TD)‐LWPLS, (C) frustratingly easy domain adaptation (FEDA)‐LWPLS and (D) the proposed method

Thus, the *r* ^2^ and RMSE values of the test data predicted using each method are presented in Table 3. Using the proposed method, the largest *r* ^2^ value and the smallest RMSE were obtained, thereby indicating that this method exhibited the highest prediction accuracy. Using the traditional methods, *r* ^2^ was negative and so, it was apparent that y could not be predicted; however, it was found that the prediction accuracy could be improved through the use of the proposed method and by the automatic decision regarding whether or not to transfer the SDs appropriately. Indeed, using this method, it was confirmed that y values could be predicted with good accuracy. Figure 3 shows the variation in the actual and predicted y values over time. From the results of TD‐LWPLS, which was the only traditional method to give a positive *r* ^2^ value, the variation in the measured y values could not be represented with the predicted y values. This was attributed to the fact that there were only 22 samples of training data, and so only a small range of y values could be predicted. Although the traditional methods cannot handle the variation in y over time, use of the proposed method allowed a wide range of y values to be predicted, and the proposed adaptive soft sensor appeared able to follow this variation in y. Thus, through a combination of TL and EL, it is possible to predict a wide range of y values while reducing the prediction y errors. It was, therefore, confirmed that the proposed method was able to appropriately predict y values, even for grades with a small number of samples.

**TABLE 3:** Obtained *r* ^2^ and root‐mean‐squared error values (RMSE) values for the test data during the generation of products that have been produced in small quantities in the past (case study 2)

**Method**	***r* ^2^**	**RMSE**
SD‐LWPLS	−3	1.279
TD‐LWPLS	0.125	0.598
FEDA‐LWPLS	−0.09	0.666
Proposed method	0.274	0.545

Abbreviations: FEDA, frustratingly easy domain adaptation; LWPLS, locally weighted partial least squares; SD, source domain; TD, target domain.

### 3.3 Case study 3: production of products of a new grade that has never been produced in the past

In this case study, it was assumed that we began the production of a new grade that had never been produced in the past. For this purpose, samples of the five grades employed in case study 1 were used as the SDs. Since no training data were available for the TD, only SD‐LWPLS, FEDA‐LWPLS and the proposed method were compared.

Thus, the *r* ^2^ and RMSE values of the test data predicted by each method are listed in Table 4, where it can be seen that the proposed method showed the best prediction accuracy with the highest *r* ^2^ value and the lowest RMSE. In addition, our results indicated that the traditional FEDA‐LWPLS (TL) method was unable to predict the y values, as confirmed by the negative nature of *r* ^2^. When predicting a new grade, adaptive modelling using other grades combined with TL fails when the number of samples increases one by one in the target grade. However, it should be noted that even in this situation, the proposed method was able to reduce the average y error by 44%.

**TABLE 4:** Obtained *r* ^2^ and root‐mean‐squared error values (RMSE) values for the test data during the generation of products of a new grade that have not been produced previously (case study 3)

**Method**	***r* ^2^**	**RMSE**
SD‐LWPLS	0.326	0.787
FEDA‐LWPLS	−0.02	0.967
Proposed method	0.678	0.544

Abbreviations: FEDA, frustratingly easy domain adaptation; LWPLS, locally weighted partial least squares; SD, source domain.

**FIGURE 4:**
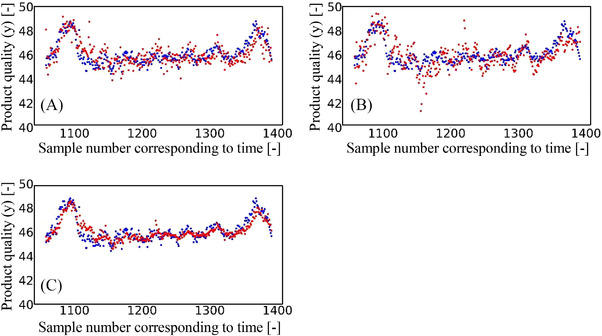
Variation in *y* overtime during the generation of products of a new grade that has not been produced in the past (case study 3). The blue and red points represent the actual and predicted *y* values, respectively. (A) source domain–locally weighted partial least squares (SD‐LWPLS), (B) frustratingly easy domain adaptation (FEDA)‐LWPLS and (C) the proposed method

Finally, Figure 4 shows the variation in the actual and predicted y values over time. More specifically, in the SD‐LWPLS and FEDA‐LWPLS methods, the variation of the predicted y value was substantial, which resulted in large y errors. On the other hand, using the proposed method, the variation of the predicted y value was relatively suppressed, and the y values were accurately predicted. These results, therefore, indicate that the proposed method improved the prediction accuracy through the use of samples of the target grades and samples of the appropriate other grades, while also employing EL with multiple adaptive soft sensors based on the TL approach. Importantly, it was also confirmed that the proposed method is able to accurately predict the y values even when the production of a new grade is started.

## 4 CONCLUSION

In this study, an adaptive soft sensor combining TL and EL was developed to predict the objective process variables (i.e. the y values) with high accuracy in a plant, where multiple product grades are produced and multiple process states exist. In the proposed method, a dataset of a target grade was used as the TD, while the datasets of the other grades were employed as the SDs. Subsequently, an adaptive soft sensor was constructed using the LWPLS approach for each SD and for each number of samples to be used as an SD. For every LWPLS model, as a greater number of TD samples were accumulated, the SDs were no longer employed. In addition, by introducing EL, multiple LWPLS models were used in a comprehensive manner to predict the y values. Importantly, using the proposed method, it was possible to predict y values in a target grade while appropriately transferring the information to other grades. The proposed method was validated using data measured in an actual incineration plant, and it was confirmed that the prediction accuracy was improved compared to that of traditional methods, such as the LWPLS model without SDs, the LWPLS model with SDs and the LWPLS model with SDs using TL. Furthermore, the proposed method was found to accurately predict y values in three different scenarios: (1) where products are produced while switching their grades, (2) in the case of a grade that has been produced in small quantities in the past and that is produced again and (3) in the case of a new grade that has never been produced in the past but is newly produced. Overall, the obtained results indicated that the proposed method could accurately predict y values using LWPLS and EL while appropriately transferring the information onto various grades. The combination of adaptive soft sensors with the proposed method will, therefore, be expected to lead to efficiency improvements in the areas of process control and process management.

## CONFLICT OF INTEREST

The authors declare that they have no known competing financial interests or personal relationships that could have influenced the work reported in this study.

## AUTHOR CONTRIBUTIONS

Nobuhito Yamada: conceptualization, methodology, software, validation, investigation, resources, data curation, writing – original draft and visualization.

Hiromasa Kaneko: conceptualization, formal analysis, resources, data curation, writing – review and editing, visualization and supervision.

## Data Availability

Data sharing is not applicable – no new data are generated.
